# Second delivery rates and recurrence of adverse pregnancy outcomes in women with systemic lupus erythematosus: a nationwide population-based cohort study

**DOI:** 10.1093/rheumatology/keag243

**Published:** 2026-05-07

**Authors:** Aleksandra Antovic, Ngoc V Nguyen, Iva Gunnarsson, Anna Sandström, Elizabeth V Arkema

**Affiliations:** Department of Medicine Solna, Division of Rheumatology and Rheumatology, Karolinska Institutet, Karolinska University Hospital, Stockholm, Sweden; Department of Medicine Solna, Clinical Epidemiology Division, Karolinska Institutet, Stockholm, Sweden; Department of Medicine Solna, Division of Rheumatology and Rheumatology, Karolinska Institutet, Karolinska University Hospital, Stockholm, Sweden; Department of Medicine Solna, Clinical Epidemiology Division, Karolinska Institutet, Stockholm, Sweden; Department of Women’s Health, Division of Obstetrics, Karolinska University Hospital, Stockholm, Sweden; Department of Medicine Solna, Clinical Epidemiology Division, Karolinska Institutet, Stockholm, Sweden

**Keywords:** systemic lupus erythematosus (SLE), adverse pregnancy outcomes (APOs), two consecutive pregnancies, population-based cohort study

## Abstract

**Objectives:**

To assess the rate of a second delivery and the recurrence of adverse pregnancy outcomes (APOs) in women with SLE compared with the general obstetric population.

**Methods:**

This nationwide population-based cohort study used Swedish registers to identify women with SLE who had a first singleton liveborn delivery after SLE diagnosis. Up to ten non-SLE comparators were matched on birth year and residence. Second delivery rates were evaluated among women whose first delivery occurred during 2003–2020, with follow-up for second deliveries until the end of 2021. Recurrence of ≥1 APO, preeclampsia and preterm delivery was assessed among women with two deliveries during 2003–2022. Cox proportional hazards models estimated hazard ratios (HRs) for second delivery, and modified Poisson models estimated risk ratios (RRs) for APO recurrence, adjusted for maternal age, education, BMI, smoking, APOs and caesarean section.

**Results:**

Second delivery rates were analysed in 543 women with SLE and 17 218 comparators. Over a median follow-up of 2.3 years, incidence rates of second delivery were 156 and 176 per 1000 person-years in women with and without SLE, respectively (adjusted HR 0.89, 95% CI 0.80–0.99). The adjusted HR was 0.78 (0.63–0.96) among women with ≥1 APO in the first pregnancy. Women with SLE had a higher risk of recurrence of APOs, including preeclampsia (RR 1.38), preterm delivery (RR 2.31) and any APO (RR 1.54).

**Conclusion:**

Women with SLE have a lower rate of a second delivery and a higher recurrence risk of APOs, highlighting the need for individualized reproductive counselling and close monitoring.

## Graphical abstract

**Graphical Abstract:**
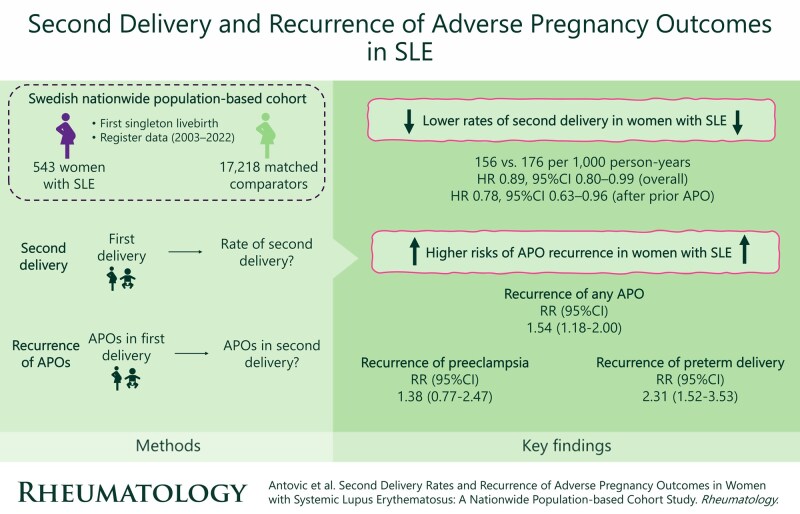

Rheumatology key messages

- Women with systemic lupus erythematosus (SLE) are less likely to have a second pregnancy, particularly following adverse outcomes in the first.
- Women with SLE have a substantially higher risk of recurrent adverse pregnancy outcomes, including preeclampsia and preterm birth.
- These findings highlight the need for individualized reproductive counselling and close monitoring before and during pregnancy.

## Introduction

Systemic lupus erythematosus (SLE) primarily affects women of childbearing age and represents a major risk factor during pregnancy. Women with SLE face an increased risk of serious adverse pregnancy outcomes (APOs), including preeclampsia, foetal intrauterine growth restriction, premature birth and foetal loss [1–4]. Additionally, pregnancy may worsen disease symptoms, triggering SLE flares and causing permanent organ damage, which significantly contributes to maternal morbidity [2, 5, 6].

Health care professionals have historically discouraged pregnancies in women with SLE due to possible maternal and foetal complications. However, advances in preconception, prenatal and perinatal care have led to safer and more frequent pregnancies among SLE. At the same time, fertility rates have declined in the general population [7], and women who experience complications during their first delivery are less likely to have subsequent pregnancies [8]. Given the elevated risk of pregnancy complications in women with SLE, it is plausible that they may have fewer subsequent pregnancies, potentially to a greater extent than women in the general population. Understanding this issue is clinically important to support family planning counselling for women with SLE, yet existing evidence remains scarce.

In the general population, APOs are more likely to recur in subsequent pregnancies following a previous APO [9, 10]. Among women with SLE, the risk of APOs in both first and subsequent pregnancies is higher than in the general population [11–13]. However, data on the recurrence risk of APOs in SLE, especially in comparison with the general obstetric population, remain limited [13]. A study using data from the Norwegian Medical Birth Registry during 1967–1995 reported higher recurrence risks of preeclampsia, preterm delivery, caesarean delivery and low birth weight in the second pregnancy in women with *vs* without rheumatic diseases, including SLE [14]. However, more recent and granular data are needed as disease characteristics and management practices have evolved.

The limited evidence on the rate of subsequent deliveries and the recurrence risk of APOs in the second pregnancy in women with SLE represents a clinically significant knowledge gap, particularly in the context of reproductive counselling for women with prior APOs. It may be hypothesized that the experience of a previous APO could influence some women with SLE to avoid subsequent pregnancies due to perceived or actual risks. Therefore, we conducted this nationwide cohort study to assess the second delivery rates and recurrence of APOs in the second pregnancy among women with SLE compared with those in the general obstetric population.

## Methods

### Study population and data sources

**Figure 1:**
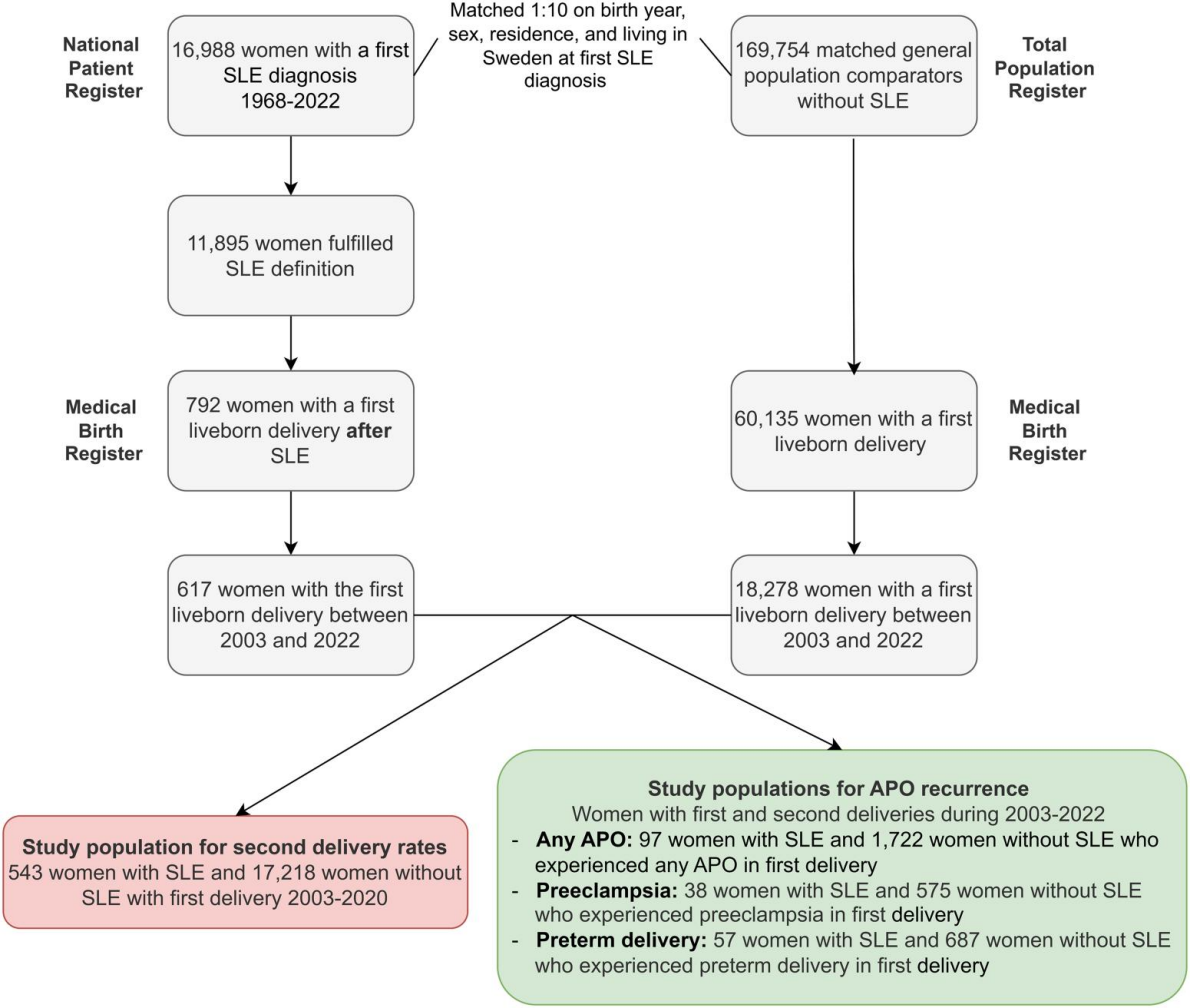
Selection of women with and without systemic lupus erythematosus who had their first liveborn delivery between 2003 and 2022 in Sweden

We conducted a nationwide register-based cohort study in Sweden. For second delivery analyses, we included women with and without SLE who had their first singleton liveborn delivery between 1 January 2003 and 31 December 2020, and followed them until 31 December 2021, for a subsequent delivery. For analyses of APO recurrence, we included a subset of women with and without SLE who had both a first and second delivery during 2003–2022 and who experienced an APO in their first delivery. Fig. 1 shows the study population selection for two aims.

Women with SLE were identified from the Swedish National Patient Register, which has nationwide data on hospitalizations since 1987 and outpatient specialist visits since 2001. SLE was defined as ≥2 visits on different dates listing an ICD code for SLE (ICD-9: 710A; ICD-10: M32, excluding drug-induced SLE M32.0). At least one of these two visits had to be with a relevant specialist (rheumatology, dermatology, nephrology, internal medicine or paediatrics). This identification method has shown good accuracy [15, 16]. For each woman with SLE, up to ten comparator women without SLE were randomly selected from the general population in Sweden, matched on birth year, residence and living in Sweden at the time of the first SLE visit of the corresponding case.

Using the unique personal identity number, women with and without SLE were linked to the Swedish Medical Birth Register to identify their deliveries. The register contains data on >98% of all deliveries in Sweden since 1973, capturing all live births regardless of gestational age and stillbirths from 28 weeks of gestation before July 2008 and from 22 weeks thereafter [17]. Because the register records only completed pregnancies, the term *pregnancy* in this study refers specifically to completed pregnancies resulting in a delivery. We required women with SLE to fulfil the SLE definition before the first date of the last menstrual period (LMP date) to ensure the women have SLE before pregnancy, while pregnancies in women without SLE were not required to occur before the matching date. The LMP date was calculated by subtracting gestational age at birth from the delivery date. Gestational age was determined by ultrasound (most preferred when available), by the last menstrual period or by clinical notes in neonatal records (least preferred).

### Outcome assessment

For second delivery rates, women were followed up from one day after the first delivery date until the LMP date of the second pregnancy, death, emigration or the end of follow-up (31 December 2021), whichever came first.

For recurrence analyses, we examined the following APOs: preeclampsia, preterm delivery and a composite outcome of any APO, defined as at least one of the following conditions: preeclampsia, preterm delivery, small-for-gestational-age (SGA) birth, gestational hypertension, gestational diabetes or placental abruption. We examined both specific APO recurrence (e.g. preeclampsia, preterm delivery) and recurrence of any APO because APOs are closely interconnected and women may experience a different APO in the second pregnancy than the one they had in the first pregnancy and/or delivery. Recurrence of SGA birth was not analysed due to small numbers (*n* < 5).

Recurrence was defined as the occurrence of the outcome of interest in the second delivery among women who experienced any APO, preeclampsia or preterm delivery in the first delivery. Accordingly, three analytical populations were derived: (i) women with preeclampsia in their first delivery; (ii) women with preterm delivery in their first delivery; and (iii) women with any APO in their first delivery, all restricted to those who had a second delivery (Fig. 1). Within each analytical population, we examined the risk of preeclampsia, preterm delivery and any APO in the second delivery.

For preeclampsia, we included diagnoses of preeclampsia, eclampsia and pre-existing hypertension with superimposed preeclampsia from gestational week 20 + 0 to six weeks post-partum. Until 2019, preeclampsia was defined as hypertension (>140/90 mmHg) in combination with excessive proteinuria [18]. In October 2019, according to the Swedish Society of Obstetrics and Gynaecology guideline, the criteria changed so that proteinuria became no longer obligatory if other organ impairment was present [19]. Preeclampsia diagnosis was identified from the Medical Birth Register (requiring ≥1 ICD code among maternal diagnoses) or National Patient Register (≥1 ICD-coded hospitalization or ≥2 ICD-coded outpatient visits). Preterm delivery was defined as delivery before gestational week 37 + 0. SGA birth was defined as birthweight below two standard deviations of sex-specific mean weight per gestational age [20]. Definitions of gestational hypertension, gestational diabetes and placental abruption are provided in Supplementary Table S1.

### Other variables

We examined maternal characteristics at the first pregnancy, including maternal age, education level, country of birth, first-trimester smoking, first-trimester body mass index (BMI; calculated from maternal weight in early pregnancy and height), as well as mode of delivery (caesarean delivery *vs* vaginal). We also calculated the interpregnancy interval, defined as the time between the first delivery date and the second pregnancy’s LMP.

Among women with SLE who had both a first and a second delivery, we described medical treatments before pregnancy (defined as ≥1 dispensed prescription during one year before LMP) and during pregnancy (defined as ≥1 dispensed prescription between LMP and delivery date). Medications included glucocorticoids, antimalarials, immunosuppressants, low-dose aspirin (ASA), heparin and low-molecular-weight heparin (LMWH). Medication dispensation data were obtained from the Prescribed Drug Register, which captures all dispensed prescribed medications in Sweden since July 2005. Consequently, medication information was available for pregnancies with deliveries between 2007 and 2022.

### Statistical analysis

Continuous variables are expressed as mean (± standard deviation, SD) and categorical variables are expressed as count (percentage). We used Cox proportional hazard models to estimate the hazard ratios and 95% confidence intervals (HR 95%CI) for having a second delivery, comparing women with and without SLE. The models were adjusted for maternal age, education, income, country of birth, first-trimester smoking, first-trimester BMI, caesarean delivery, preeclampsia, preterm delivery, SGA birth and other APOs at first delivery. Additionally, we stratified the analysis by the presence or absence of any APOs in the first delivery.

Modified Poisson models were used to estimate the risk ratios (RR 95%CI) for APO recurrence, comparing women with and without SLE, adjusted for maternal age, education, country of birth, first-trimester smoking, first-trimester BMI, caesarean delivery in first delivery and interpregnancy interval. Additionally, we compared medication treatment between the first and second deliveries using McNemar’s test. This study was approved by the Swedish Ethical Review Authority (2021–01148). All analyses were performed with R version 4.3.

## Results

We included 543 women with SLE and 17 218 women without SLE whose first deliveries occurred during 2003–2020 for the analysis of the second delivery rate. Different analytical populations were derived for recurrence analyses depending on the specific APOs (Fig. 1).

General characteristics of primiparous women with and without SLE (2003–2020) are presented in Table 1. The mean maternal age was about 30 years and was similar between women with and without SLE. Compared with women without SLE, those with SLE were less likely to report smoking during the first trimester and had a slightly lower proportion of first-trimester obesity (BMI ≥30 kg/m^2^). They were also more likely to have ≥13 years of education. Caesarean delivery and all investigated APOs were more common in women with SLE.

**Table 1:** Maternal characteristics at first delivery in women with and without systemic lupus erythematosus, Sweden, 2003–2020.

Maternal characteristics at first delivery	Women with SLE (N = 543)	**Women without SLE (N = 17** **218)**
Age, mean (SD)	30.5 (4.6)	30.0 (5.0)
Nordic country of birth, *n* (%)	471 (86.7)	14 706 (85.4)
First-trimester smoking, *n* (%)	19 (3.5)	897 (5.2)
First-trimester BMI ≥30 kg/m^2^, *n* (%)	50 (9.2)	1718 (10.0)
≥13 years of education, *n* (%)	322 (59.3)	9150 (53.1)
Annual income level ≥300 000 Swedish krona, *n* (%)	187 (34.4)	6339 (36.8)
Caesarean delivery, *n* (%)	177 (32.6)	3274 (19.0)
Elective, *n* (%)	96 (17.7)	1263 (7.3)
Emergency, *n* (%)	81 (14.9)	2011 (11.7)
Adverse pregnancy outcomes		
Preeclampsia, *n* (%)	65 (12.0)	853 (5.0)
Preterm delivery, *n* (%)	97 (17.9)	1004 (5.8)
Spontaneous onset, *n* (%)	38 (7.0)	703 (4.1)
Medically indicated onset, *n* (%)	59 (10.9)	301 (1.7)
Small for gestational age birth, *n* (%)	55 (10.1)	558 (3.2)
Other APOs,^a^ *n* (%)	24 (4.4)	634 (3.7)

a Any of gestational hypertension, gestational diabetes or placental abruption.

APO: adverse pregnancy outcome; BMI: body mass index; SD: standard deviation; SLE: systemic lupus erythematosus.

### Second delivery rates in women with and without SLE

Over 2.3 years of median follow-up (interquartile range 1.4–4.7 years), 348 women with SLE and 12 632 women without SLE had a second delivery (incidence rate 156 per 1000 person-years for SLE and 176 per 1000 person-years for non-SLE) (Table 2). The age-adjusted rate difference between SLE and non-SLE was –18 (–40 to +4) delivery per 1000 person-years. The fully-adjusted HR was 0.89 (95%CI 0.80–0.99). Regardless of SLE status, women who experienced any APOs in the first delivery were less likely to have a second delivery compared with those without any APOs in the first delivery. The second delivery HRs between SLE and non-SLE in stratified analysis were 0.78 (0.63–0.96) in women who experienced any APOs in the first delivery and 0.94 (0.83–1.06) in women who did not experience any APOs in the first delivery. The mean interpregnancy interval was 2.34 years for women with SLE and 2.30 years for those without SLE.

**Table 2:** Delivery rates in women with and without systemic lupus erythematosus, overall and stratified by adverse pregnancy outcomes in first pregnancy, Sweden, 2003–2020.

Population	**Second delivery rate** ^a^		**Age-adjusted rate difference** ^a^ **(95%CI)**	**Fully adjusted HR** ^b^ **(95%CI)**
	Women with SLE	Women without SLE		
Overall	156	178	–18 (–40 to +4)	0.89 (0.80–0.99)
With at least one APO in the first pregnancy^c^	114	148	–36 (–70 to –2)	0.78 (0.63–0.96)
Without any APO in the first delivery^c^	180	183	+1 (–28 to +31)	0.94 (0.83–1.06)

a Incidence rates and rate differences were presented as the number of second pregnancies per 1000 person-years.

b Adjustment included maternal age, education, income, country of birth, first-trimester smoking, first-trimester body mass index, caesarean delivery, preeclampsia, preterm delivery, small for gestational age birth and other adverse pregnancy outcomes (gestational hypertension, gestational diabetes, placental abruption).

c APOs used to stratify the analysis included preeclampsia, preterm delivery, small for gestational age birth, gestational hypertension, gestational diabetes and placental abruption. Hence, the adjustment included all the above variables, except for these APOs.

RR 95%CI: risk ratio and 95% confidence interval; SLE: systemic lupus erythematosus.

### Recurrence risk of APOs

The recurrence risk of any APO, preeclampsia and preterm delivery separately was consistently higher in women with *vs* without SLE (Table 3). For instance, among 97 women with SLE who experienced any APO in the first delivery, 41 also had at least one APO in their second delivery (recurrence risk 42.3%), compared with 464 of 1722 women without SLE (26.9%) (RR 95%CI 1.54 [1.18–2.00]). The specific recurrence risk of preeclampsia following preeclampsia was 31.6% in SLE and 19.8% in non-SLE (1.38 [0.77–2.47]). The specific recurrence risk of preterm delivery following a first preterm delivery was 36.8% in SLE and 15.6% in non-SLE (2.31 [1.52–3.53]). The association between SLE and APO recurrence appeared to be stronger for preterm delivery compared with preeclampsia or any APO (i.e. consistently higher RRs for preterm delivery). Some of the 95% CIs were wide and indicated a possibility of a null association, possibly due to the low number of recurrence events.

**Table 3:** Recurrence risk of any adverse pregnancy outcome, preeclampsia and preterm delivery in women with and without systemic lupus erythematosus, Sweden, 2003–2022

Population (N women with and without SLE)	Outcome in second delivery	Recurrence risk, *n* (%)		**Adjusted RR** ^a^ **(95% CI)**
		Women with SLE	Women without SLE	
Any APO in first delivery (97 SLE and 1722 non-SLE)	Any APO	41 (42.3)	464 (26.9)	1.54 (1.18–2.00)
	Preeclampsia	17 (17.5)	147 (8.5)	1.82 (1.10–3.01)
	Preterm delivery	27 (27.8)	151 (8.8)	2.97 (2.02–4.38)
Preeclampsia in first delivery (38 SLE and 575 non-SLE)	Any APO	16 (42.1)	201 (35.0)	1.08 (0.70–1.68)
	Preeclampsia	12 (31.6)	114 (19.8)	1.38 (0.77–2.47)
	Preterm delivery	9 (23.7)	37 (6.4)	3.21 (1.48–6.98)
Preterm delivery in first delivery (57 SLE and 687 non-SLE)	Any APO	26 (45.6)	181 (26.3)	1.47 (1.04–2.08)
	Preeclampsia	10 (17.5)	51 (7.4)	1.64 (0.80–3.35)
	Preterm delivery	21 (36.8)	107 (15.6)	2.31 (1.52–3.53)

a Adjustment included maternal age, education, income, country of birth, first-trimester smoking, first-trimester body mass index, caesarean delivery and pregnancy interval.

RR 95%CI: risk ratio and 95% confidence interval; SLE: systemic lupus erythematosus.

### Treatments for women with SLE with two consecutive pregnancies

During 2007–2022, 279 women with SLE had both a first and a second delivery. As shown in Supplementary Table S2, the use of most treatment modalities before and during pregnancy was not statistically different between first and second deliveries. However, there was an increase in the use of LMWH and low-dose aspirin, both before and during pregnancy, while the use of immunosuppressants decreased between the first and second pregnancies.

## Discussion

In this nationwide population-based cohort study, we have investigated second delivery rates and recurrence of APOs in the second delivery in women with SLE in comparison to women without SLE. APOs were more frequent in the first delivery among women with SLE, and these women had a lower rate of a subsequent delivery compared with women without SLE who also experienced an APO in the first delivery. This particularly relates to women who had experienced an APO in their first pregnancy, suggesting that prior complications may influence reproductive decisions in SLE. In contrast, women with SLE whose first delivery was uncomplicated had similar rates of second delivery as women in the general population.

Further, women with SLE had an increased risk of recurrent preeclampsia and preterm delivery in their second pregnancy compared with women without SLE. About a third of women with a first delivery affected by preeclampsia or preterm delivery go on to have a second delivery with preeclampsia or preterm delivery. Although the number of recurrent SGA births was too small for formal analysis, our results are consistent with previous literature suggesting increased risk of foetal growth restriction in SLE pregnancies. Our findings underscore the cumulative burden of reproductive risks in women with SLE, even in the context of improved prenatal counselling and treatment strategies.

Data from the Swedish Medical Birth Register (1987–2004) indicated a substantially increased recurrence risk for preeclampsia among women in the general population with a history of preeclampsia, reaching 14.7% in the second pregnancy following a first affected pregnancy [10]. Further, the proportion of women who had a subsequent pregnancy was 4–5% lower if the first pregnancy was complicated by preeclampsia, and >10% lower when preeclampsia was accompanied by preterm delivery. This is in line with our findings showing that irrespective of SLE status, women who experienced at least one APO at the first delivery had a lower second delivery rate compared with those without APOs. In our study, the risk of recurrent preeclampsia was 66% higher in women with SLE (aRR 1.66; 95% CI 0.96–2.90). The point estimate suggests a clinically relevant increase. Although the confidence interval includes the null value, possibly due to a small number of recurrent events, most of the interval lies above 1.0, suggesting an increased risk.

The most pronounced association in our study was observed for preterm delivery, where the recurrence risk was more than twice as high in SLE compared with controls (aRR 2.32; 95% CI: 1.55–3.46).The strong recurrence pattern suggests that once preterm delivery occurs, women with SLE remain at high risk of repetition, emphasizing the need for close monitoring and proactive management in later pregnancies.

Taken together, these results indicate that APOs tend to cluster across pregnancies in women with SLE, with preterm delivery showing the most robust recurrence pattern. This underlines the value of using pregnancy history as a simple but powerful marker of future risk. Identifying women with prior APOs may help guide surveillance intensity and preventive strategies in subsequent pregnancies.

Previous studies on recurrent APOs in women with SLE are scarce and were conducted prior to the current recommendations for the management of pregnancy in SLE patients [21, 22]. Today, most women with SLE can achieve successful pregnancies, if appropriate measures are implemented to minimize the risk of APOs. Pharmacological management primarily relies on hydroxychloroquine (HCQ) and low-dose aspirin (LDA) to reduce disease activity and prevent PE. While HCQ consistently lowers disease flares [23–25], its protective effect against preeclampsia remains uncertain [26, 27]. However, a recent Swedish population-based study reported a lower risk of preeclampsia among SLE pregnancies exposed to HCQ early in gestation [28]. In the present study, HCQ use was around 60% and remained unchanged in subsequent pregnancies, indicating potential for improved adherence. The protective role of LDA against preeclampsia is established, particularly in women with lupus nephritis and/or the presence of antiphospholipid antibodies (aPL) [29]. However, a prior retrospective cohort study of 144 consecutive SLE pregnancies showed persistently elevated risk of APOs in SLE, despite low disease activity before, during and after pregnancy and irrespectively of aPL status [30]. Around one-third of women included in our study received LMWH, serving as a proxy for aPL positivity or antiphospholipid syndrome (APS), which is often underreported in national registries but presents an independent risk factor for preeclampsia, repeated foetal loss, SGA and preterm delivery [2]. We report here increased administration of LDA and LMWH between pregnancies, suggesting an improved implementation of preventive strategies in clinical practice over time.

Our study has several limitations. We did not have data on preconception disease activity, flares and organ involvement, which may influence both pregnancy outcomes and decisions regarding future pregnancies. This is particularly related to SLE patients with renal involvement at high risk for APOs. We could not examine all pregnancy complications, such as miscarriages (not available in the Medical Birth Register) and intrauterine foetal deaths or stillbirths (too few to analyse, *n* < 5), and the results therefore apply only to the selected APOs analysed. In addition, the number of recurring events for some APOs was small, resulting in wide confidence intervals. Our recurrence risk results are based on the inherent selection of women who had two liveborn deliveries who are potentially healthier than women who did not, and generalization of our results should keep this in mind. Still, we have used nationwide register data in a population with universal access to health care, an accurate SLE definition, as well as longitudinal follow-up of pregnancy outcomes.

Future studies with detailed data on antibody status, disease activity and manifestations, treatment indication and dosing are needed to clarify how anticoagulant and immunosuppressive therapies between consecutive pregnancies influence the risk of recurrent APOs in women with SLE.

To conclude, elevated recurrence risks of preeclampsia and preterm delivery in pregnant women with SLE are concerning, particularly since both conditions are linked to higher maternal and neonatal morbidity and may influence the decisions regarding future family planning. Our study shows that women with SLE who experienced preeclampsia or preterm birth in their first pregnancy were up to twice as likely to experience the same complication again compared with women without SLE. These findings highlight the importance of close monitoring, individualized counselling and tailored risk assessment before and during pregnancy in women with SLE.

## Supplementary Material

keag243_Supplementary_Data

## Data Availability

Data are not publicly available due to legal restrictions. Requests for study data can be sent to the corresponding author.
