# Supplementary material for: Second delivery rates and recurrence of adverse pregnancy outcomes in women with systemic lupus erythematosus: a nationwide population-based cohort study
Source: Rheumatology (Oxford). 2026 May 7;65(6):keag243. doi: 10.1093/rheumatology/keag243 (PMC13231447; doi:10.1093/rheumatology/keag243)
Supplement: keag243_Supplementary_Data [file keag243_supplementary_data.docx]

SUPPLEMENTARY MATERIALS

**Supplementary Table S1.** Definitions of all variables used in the study

| **Variable** | **Data source & Definition** | **ICD/ATC code and/or categorization** |
| --- | --- | --- |
| ***Patients and pregnancies with prevalent SLE*** | | |
| SLE | NPR: ≥2 ICD-coded visits on different dates, ≥1 visit with a specialist typically treating SLE: rheumatology, dermatology, nephrology, internal medicine, pediatrics | ICD-8: 734.1  ICD-9: 710.0  ICD-10: M32.1-M32.9 |
| LMP | MBR: First date of the last menstrual period: Time between delivery date and gestational length at delivery |  |
| SLE pregnancy | Fulfilling SLE definition before LMP |  |
| ***Adverse pregnancy outcomes*** | |  |
| Preeclampsia | - MBR: ≥1 ICD code in the variable MDIAG, or - NPR: ≥1 ICD-coded inpatient admission or ≥2 ICD-coded outpatient visits from week 20+0 to week 6+0 post-partum - If one pregnancy has both gestational hypertension and preeclampsia, only preeclampsia is considered. | ICD10: O11, O14, O15 |
| Gestational hypertension |  | ICD-10: O13 |
| Placental abruption | - MBR: ≥1 ICD code in the variable MDIAG, or - NPR: ≥1 ICD-coded inpatient admission or outpatient visit from week 20+0 to week 6+0 post-partum | ICD-10: O45 |
| Preterm delivery overall | MBR: Delivery before 37+0 weeks of gestation | |
| Small for gestational age birth | MBR: Birthweight below two standard deviations of sex-specific mean weight per gestational age |  |
| Caesarean delivery | MBR: Both elective and emergency Caesarean |  |
| **SLE treatments** | PDR  Before pregnancy: ≥1 dispensation within one year before LMP  During pregnancy: ≥1 dispensation from LMP to delivery date | |
| Antimalarials | Chloroquine and hydroxychloroquine | P01BA01, P01BA02 |
| Systemic corticosteroids |  | H02AB01, H02AB02, H02AB04, H02AB06, H02AB07 |
| Immunosuppresants | Cyclophosphamide, azathioprine, methotrexate, ciclosporin, tacrolimus, voclosporin, sirolimus, mycophenolate mofetil | L01AA01, L04AX01, L04AX03, L04AD01, L04AD02, L04AD03, L04AH01, L04AA06 |
| Low-dose aspirin | Assessed only during pregnancy | B01AC06 |
| Heparin and low molecular weight heparins |  | B01AB01, B01AB04, B01AB05, B01AB06, B01AB07, B01AB08, B01AB10, B01AB12 |
| ***Over variables*** |  |  |
| Maternal age at delivery | MBR: Age in years | Continuous variable in years |
| Country of birth | LISA | Nordic/Non-Nordic |
| Education level in the year before the LMP year | LISA | ≤9 years, 10-12 years, ≥13 years |
| Income level in the year before the LMP year | LISA | <100, 100-299, ≥300 (x1000 SEK) |
| 1^st^ trimester smoking | MBR | Yes/No |
| 1^st^ trimester BMI | MBR: derived from height (m^2^) and weight (kg), in kg/m^2^ | - Continuous variable in kg/m^2^ - Categorical variable: underweight (<18.5 kg/m^2^), normal weight (18.5–24.9 kg/m^2^), overweight (25.0–29.9 kg/m^2^), obese (≥30.0 kg/m^2^) |
| Parity | MBR | Nulliparous/Parous |

ICD: International classification of disease; ATC: Anatomical therapeutic chemical classification; MBR: Medical Birth Register; NPR: National Patient Register; PDR: Prescribed Drug Register; LISA: Swedish Longitudinal Integrated Database for Health Insurance and Labor Market Studies; SLE: Systemic lupus erythematosus; LMP: First date of last menstrual period; BMI: Body mass index

**Supplementary Table S2.** Treatment for women with systemic lupus erythematosus during one year before pregnancy and during pregnancy in their first and second deliveries, Sweden, 2007-2022

| Drug treatment | **One year before pregnancy, n (%)** | | | **During pregnancy, n (%)** | | |
| --- | --- | --- | --- | --- | --- | --- |
|  | **1^st^ pregnancy (N=279)** | **2^nd^ pregnancy (N=279)** | p-value* | **1^st^ pregnancy (N=279)** | **2^nd^ pregnancy (N=279)** | p-value* |
| **Antimalarials** | 182 (61.3) | 186 (62.6) | 0.699 | 169 (56.9) | 181 (60.9) | 0.112 |
| **Glucocorticoids** | 149 (50.2) | 151 (50.8) | 0.896 | 150 (50.5) | 140 (47.1) | 0.282 |
| **Immunosuppressants** | 117 (39.4) | 95 (32.0) | 0.003 | 89 (30.0) | 87 (29.3) | 0.860 |
| *Azathioprine* | 91 (30.6) | 84 (28.3) | 0.310 | 85 (28.6) | 82 (27.6) | 0.710 |
| *Methotrexate* | 11 (3.7) | <5 |  | 0 | 0 |  |
| *Mycophenolate mofetil* | 27 (9.1) | 13 (4.4) | 0.011 | <5 | <5 |  |
| *Calcineurin inhibitors* | 11 (3.7) | 13 (4.4) | 0.752 | 14 (4.7) | 15 (5.1) | 1.000 |
| **Heparin and LMWH** | 16 (5.4) | 38 (12.8) | <0.001 | 75 (25.3) | 90 (30.3) | 0.018 |
| **Low-dose aspirin (75mg/day)** | 37 (12.5) | 71 (23.9) | <0.001 | 171 (57.6) | 193 (65.0) | 0.001 |

* p-values from McNemar’s test

LMWH: Low molecular weight heparin
